# Anesthesia management of atrial myxoma resection with multiple cerebral aneurysms: a case report and review of the literature

**DOI:** 10.1186/s12871-020-01055-1

**Published:** 2020-07-04

**Authors:** Ran Zhang, Zhiyu Tang, Qing Qiao, Feroze Mahmood, Yi Feng

**Affiliations:** 1 Department of Anesthesiology, Peking University People’s Hospital, No. 11 Xi Zhi Men Nan Da Jie, Xicheng District, Beijing, China; 2 Department of Anesthesiology, Beth Israel Deaconess Medical Center, Boston, USA

**Keywords:** Multiple cerebral aneurysms, Atrial myxoma, Anesthesia management

## Abstract

**Background:**

Embolic stroke is a common complication of atrial myxoma, whereas multiple cerebral aneurysms associated with atrial myxoma is rare. The pathogenesis of the cerebral vascular disease related to an atrial myxoma is still not well known, and there are no guidelines to guide treatment and anesthesia management in such patients.

**Case presentation:**

In this report, we present a 38-year-old woman with occasional dizziness and headache diagnosed as multiple cerebral fusiform aneurysms, in whom transthoracic echocardiography revealed a mass attached to the interatrial septum in the left atrium. Myxoma resection was performed in fast track cardiac surgery pathway without neurological complications, and no intervention was carried out on the cerebral aneurysms. She was discharged home 6 days after the procedure for followed-up. Furthermore, we reviewed and analyzed the literature in the PubMed and Google Scholar databases in order to conclude the optimal treatment in such cases.

**Conclusions:**

Atrial myxoma-related cerebral aneurysms are always multiple and in a fusiform shape in most occasions. Early resection of myxoma and conservative therapy of aneurysm is an optimal treatment. TEE and PbtO_2_ monitoring play an essential role in anesthesia management. Fast track cardiac anesthesia is safe and effective to early evaluate neurological function. Long term follow-up for “myxomatous aneurysms” is recommended. And outcome of most patients is excellent.

## Background

Atrial myxoma is the most common benign cardiac tumor, which represents about 50% of all primary cardiac tumors. Approximately 75% occur in the left atrium [1]. Systemic embolism due to atrial myxoma has been well documented, especially embolic stroke [2]. However, intracranial aneurysms are rarely associated to atrial myxoma [3]. We present the case of a woman with dizziness and headache whose brain computed tomography angiography (CTA) manifested multiple fusiform aneurysms, and transthoracic echocardiography revealed a mass in the left atrium.

The pathogenesis of the cerebral vascular disease related to an atrial myxoma is still not well known, and there are no guidelines to guide treatment and anesthesia management in such patients.

## Case presentation

### Case report

**Fig. 1:**
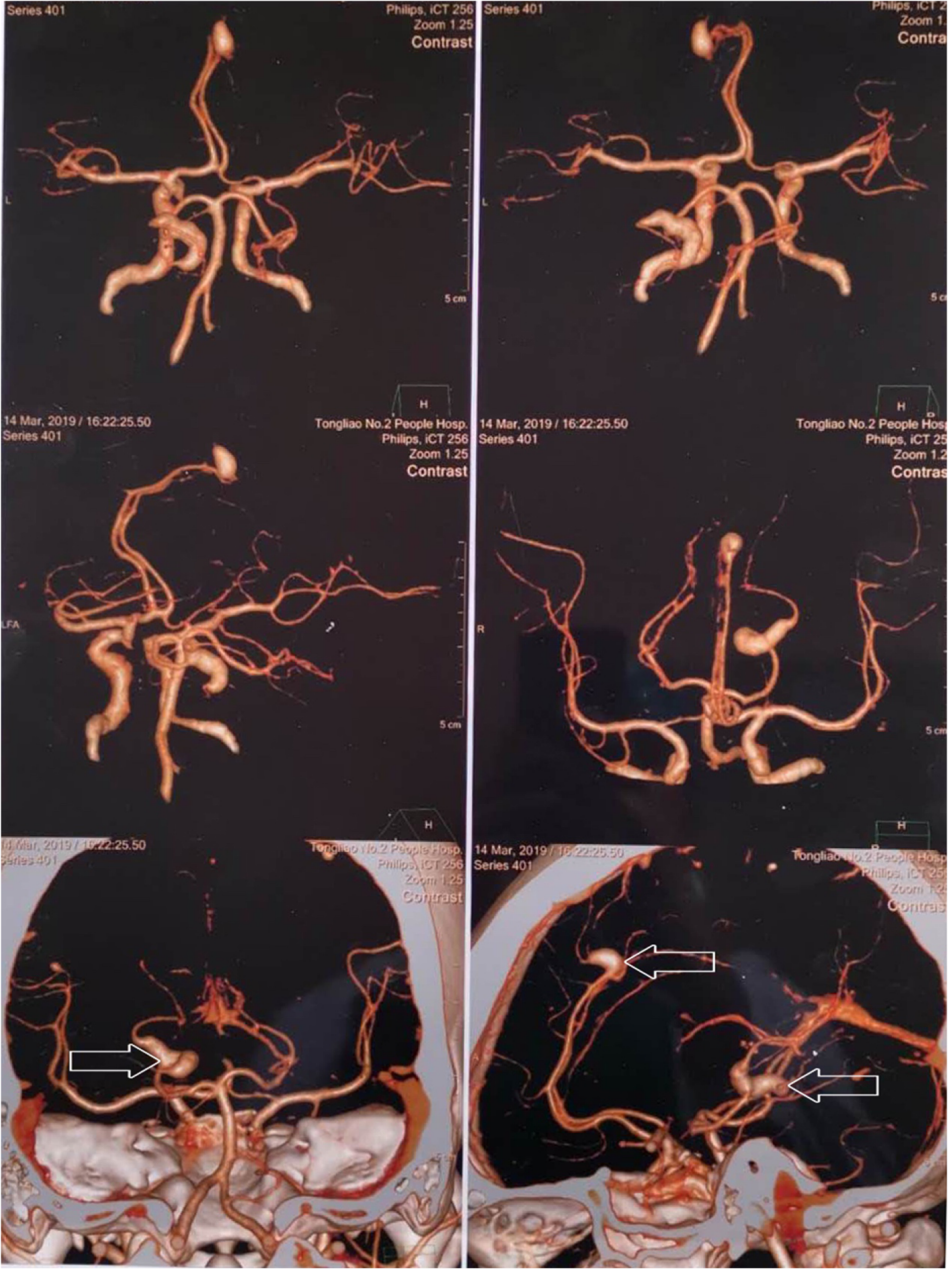
Two fusiform aneurysms located in left anterior cerebral artery and left posterior cerebral artery, with the size of 9.7 mm × 6.3 mm and 10.2 mm × 7 mm, respectively

**Fig. 2:**
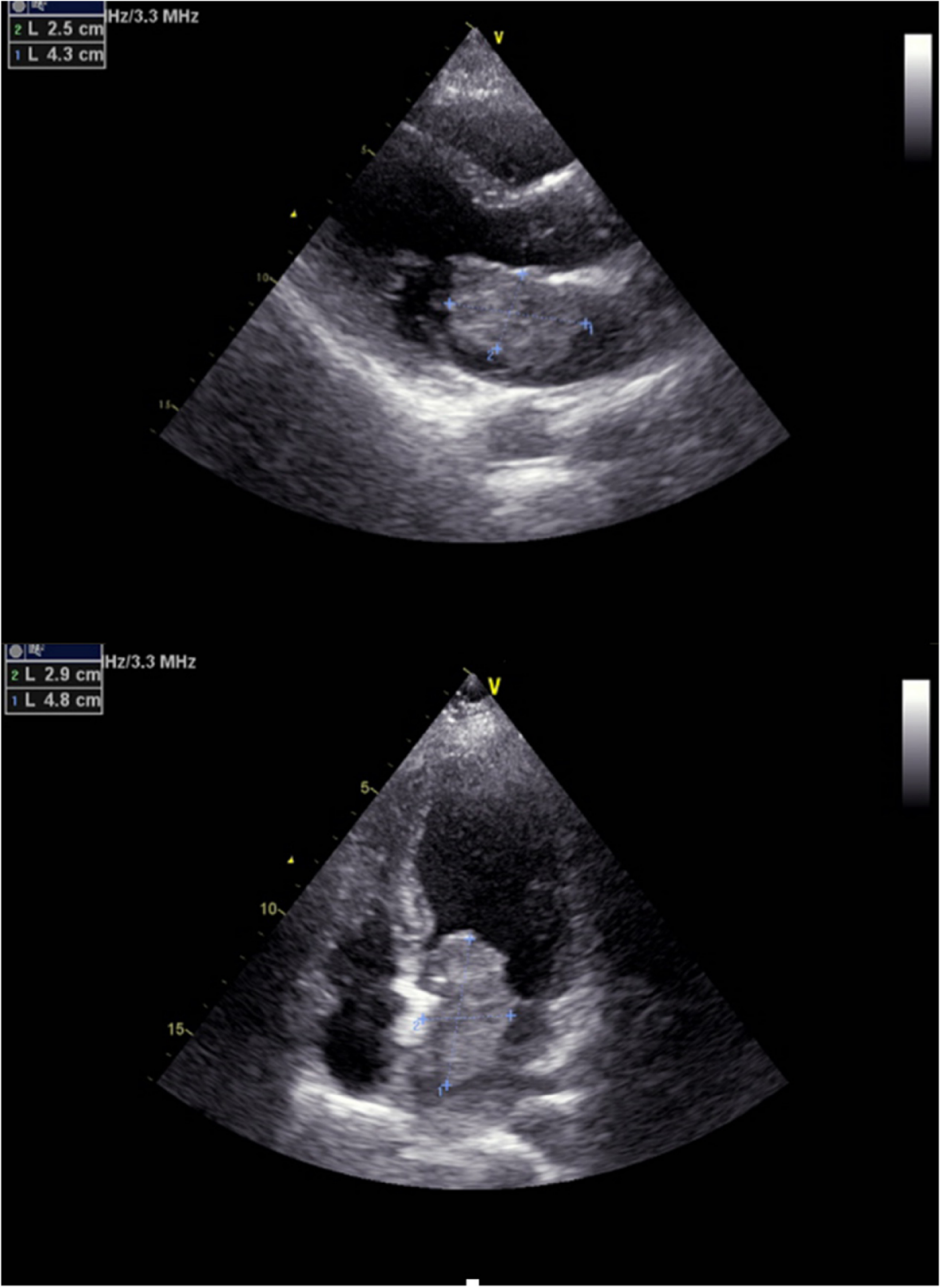
TTE revealed a 4.8 × 2.9 × 2.5 cm^3^ mass attached to the interatrial septum in the left atrium, which obstructed the mitral orifice without mitral valve regurgitation

A 38-year-old woman with no medical history presented 10 days of dizziness and headache without loss of consciousness, dysarthria, weakness, nausea, or vomiting. Neurological examination was normal. The brain CTA manifested two unruptured fusiform aneurysms, which located in left anterior cerebral artery and left posterior cerebral artery, with the size of 9.7 mm × 6.3 mm and 10.2 mm × 7 mm, respectively (Fig. 1). Furthermore, transthoracic echocardiography (TTE) revealed a 4.8 × 2.9 × 2.5 cm^3^ mass attached to the interatrial septum in the left atrium, which obstructed the mitral orifice without mitral valve regurgitation (Fig. 2).

**Fig. 3:**
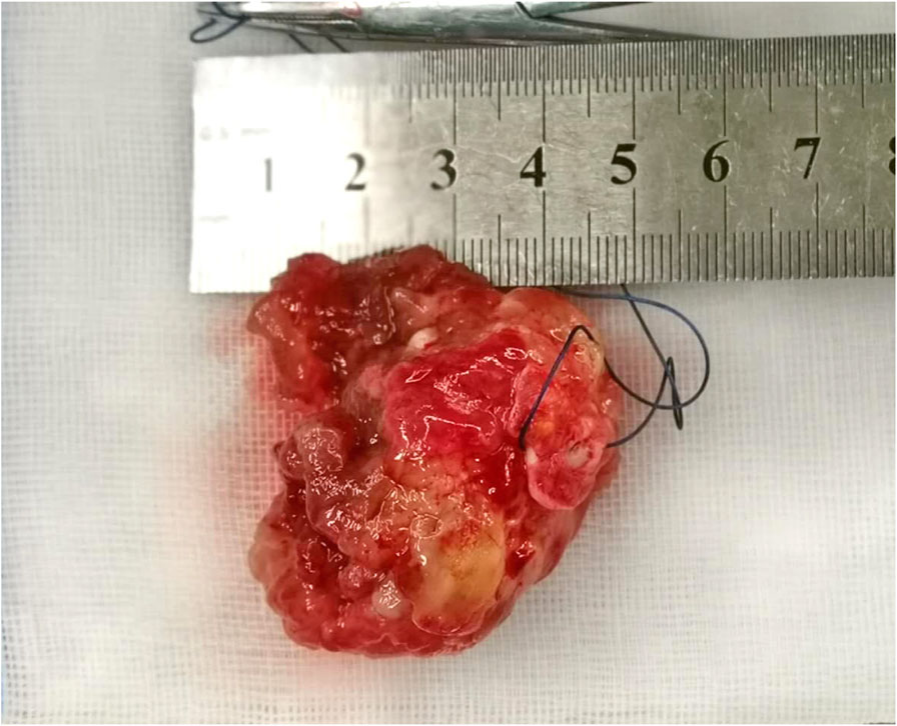
Polypoid type of atrial myxoma

**Fig. 4:**
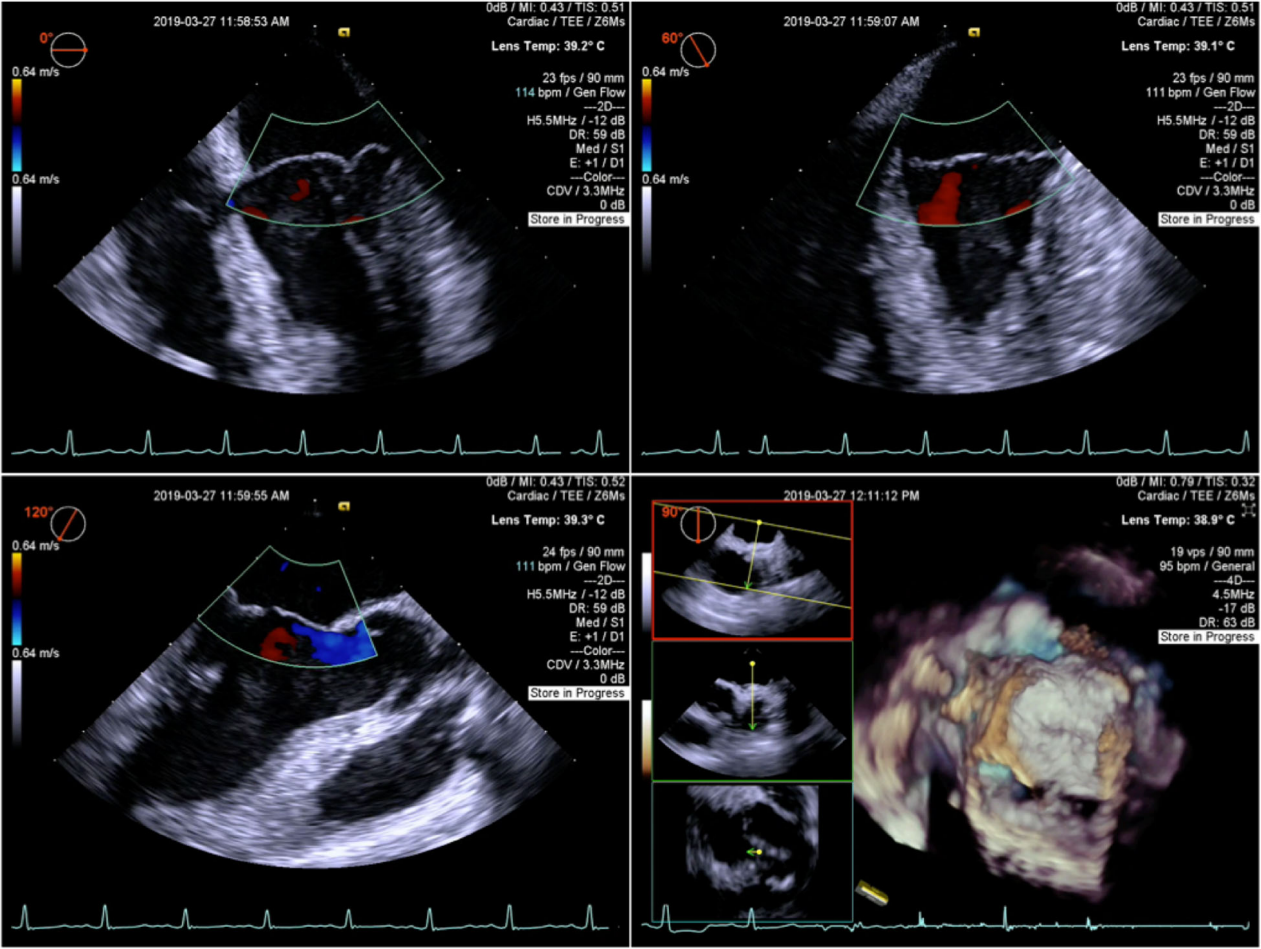
After resection of myxoma, no mitral regurgitation or shunt flow across the atrial septum was revealed by transesophageal echocardiography

According to the recommendation of multidisciplinary team (MDT), myxoma was first considered to be excised, a conservative approach was chosen for cerebral aneurysms, and the fast track cardiac surgery pathway should be performed to evaluate neurological function as soon as possible. The baseline vital signs of this patient were measured before induction of general anesthesia, in order to maintain the fluctuation range of heart rate (HR) and mean arterial pressure (MAP) within 10% throughout the perioperative period. The mass was successfully removed and histological examination confirmed a typical myxoma (Fig. 3). No mitral regurgitation or shunt flow across the atrial septum was revealed by transesophageal echocardiography (TEE) (Fig. 4). Parenchymal brain oxygen (PbtO_2_) monitoring did not change significantly throughout the procedure. The patient was transported to intensive care unit (ICU) receiving infusion of propofol. After that, continuous infusion of fentanyl (0.3μg/kg × h^− 1^) was performed to ensure analgesia and attenuate cardiovascular response to tracheal intubation. She was extubated 3 h after surgery without neurological disorder and discharged from ICU on the first day. Intravenous patient-controlled analgesia pump was employed to insure postoperative numeric rating scale (NRS) score lower than 3 (0 = No pain, 10 = worst pain imaginable) [4]. She was fully recovered and discharged home on the sixth day after surgery.

### Review and analysis of the literature

The keywords “cerebral aneurysm”, “intracranial aneurysm”, “myxoma”, and “anesthesia” were used for searching in the PubMed and Google Scholar databases. The literature written in English published from January 1966 to April 2019 was reviewed, and articles or abstracts providing the following information were included, for instance, age, gender, intervention for myxoma and aneurysm, complication, and outcome. Eventually, there were 47 reports of 49 cases and a total of 50 cases analyzed [3, 5–49]. The median age was 38 years (95%CI, 34–42), and female/male ratio was 3.17:1. Resection of atrial myxoma was performed first in 90% (45) cases. Among these, conservative therapy for cerebral aneurysm was performed in 70% (35) cases, including repeated operations of recurrence myxoma in 2 cases [33, 40]. Whereas, craniotomy for aneurysm in 3 cases [8, 11, 19], coiling for 2 cases [15, 34], radiation for 1 case [32], and cytostatic treatment for 1 case was carried out later [12]. Only one case reported craniotomy was performed first and early resection of myxoma was advised [13]. Three patients were dead in the early 1970s due to lack of knowledge and treatment [45, 46, 48]. After resection of myxoma, 13.3% (6/45) patients suffered neurological dysfunction, while acute left hemiparesis appeared during induction of anesthesia and the operation was delayed in one case [5]. Severe neurological complication appeared in one patient with chronic renal failure, who finally died of sepsis [22]. No perioperative subarachnoid hemorrhage (SAH) was reported. Except in one patient, a conservative therapy was attempted, and a myxoma was verified by autopsy with cerebral aneurysms in 1973 [45]. During follow-up period, the rates of stable and regression of aneurysm were 50% (25 cases) and 10% (5 cases) respectively, while enlargement was 10% (5 cases), and new formation was 12% (6 cases). The subgroup of 11 progressive cases was further analyzed, continuous conservative therapy was performed in 4 cases, operation was carried out in 3 cases, and radiotherapy was administered in one case. Further follow-up revealed stable or regression after the treatment. Only one patient suffered SAH [21]. Although anesthesia management was introduced in only one case, it was in craniotomy procedure [13].

## Discussion and conclusions

The incidence of primary heart tumors is less than 0.2% in patients. 75% of the tumors are benign, in which approximately 50% are myxomas [1]. Nearly three quarters of myxomas are located in the left atrium, while 15 ~ 20% are in the right atrium. Up to 20% of patients can be asymptomatic, whereas in a large case series, mitral valve obstruction, systemic emboli, and constitutional symptoms occurred [50, 51]. Systemic emboli has been well documented, especially embolic stroke [52, 53]. It was reported a villous myxoma might be associated with more chances of metastasis of myxomas, and polypoid type was the only independent predictor of systemic emboli [54]. However, cerebral aneurysms related to atrial myxoma are rare. This patient was asymptomatic with myxoma, and neurological symptoms appeared first, for instance, dizziness and headache. The myxoma was polypoid type in this case.

In 1894, Marchand first reported an interesting phenomenon that cerebral aneurysms were associated with atrial myxoma [55]. Until 2005, Sabolek demonstrated the typical manifestation of aneurysms were multiple with fusiform shape [27]. To date, only around 50 case reports written in English could be found in the literature (Table 1). However, the exact mechanism is still not clear. The hypothesis of “Metastasize and Infiltrate” was considered as an essential mechanism for cerebral aneurysm formation. Myxoma cells may metastasize to the cerebral arteries, infiltrate into the vessel wall through the vasa vasorum or endothelial, interrupt the elastic lamina, and lead to aneurysm formation. Histological examination of the excised cerebral aneurysm verified this hypothesis [29, 36, 48]. Recent reports proposed another hypothesis, which is inflammation reaction arised from myxoma. It is reported that new cerebral aneurysms can form after myxoma resection, without recurrent myxoma or embolism [56]. Some studies found that new aneurysm formed with elevated proinflammation cytokines like interleukin-6 (IL-6) after resection of myxoma [27]. What is more, IL-6 level upregulated by myxoma may contribute to aneurysm formation [57, 58]. Other researches illuminated that IL-6 could promote matrix metalloproteinases expression and activity, which enhance invasion of myxoma cells [23, 59]. Unfortunately, IL-6 level was not tested in our patient.

**Table 1:** Case reports of multiple aneurysms related to atrial myxoma

	Author	Year	Age	Gender	Procedure (myxoma, aneurysm)	Complication	Outcome
1	This case	2019	38	F	Resection, Conservative	None	Discharge 6 days postoperation and follow-up
2	Coutinho R, et al	2018	46	F	Resection, Conservative	Acute left hemiparesis during induction	Aneurysms completely regressed 18 days later and follow-up
3	Penn DL, et al	2018	12	M	Resection, Conservative	None	1 year follow-up, growth of 2 aneurysms, hybrid procedure, 43 months follow-up, unchanged
4	Flores PL, et al	2018	61	M	Resection, Conservative	None	18 months follow-up, unchanged
5			19	F	Resection, Conservative	None	5 year follow-up, several enlarged, others regressed, asymptomatic, conservative
6	Yoo HJ, et al	2018	20	F	Resection, Craniotomy later	Lost vision in right eye	Not mentioned
7	Quan K, et al	2017	49	F	Resection, Conservative	Not mentioned	Further intracranial lesions resection may be performed
8	Sveinsson O, et al	2015	19	F	Resection, Conservative	None	1 year follow-up, unchanged
9	Zheng J, et al	2015	25	F	Resection first, craniotomy 7 months later	Drowsiness and partial seizure 6 days after craniotomy	Discharge 7 days later, 2 months follow-up, unchanged
10		2015	39	F	Resection 20 years ago, Conservative	None	14 months follow-up, occastional dizziness
11	Vontobel J, et al	2015	41	F	Resection, Cytostatic treatment	None	Follow-up, decreased tracer uptake in PET, stable aneurysm sizes
12	Srivastava S, et al	2014	30	F	None, Craniotomy first	None	Discharge 7 days later, early resection of myxoma was advised
13	Xu Q, et al	2013	46	F	Resection, Conservative	None	Follow-up
14	Al-Said Y, et al	2013	67	F	Resection, Coiling 1 week later	None	1 year follow-up, unchanged
15	Kim H, et al	2012	58	M	Resection, Conservative	None	1 year follow-up, unchanged
16	KJ George, et al	2012	45	F	Resection, Conservative	None	Discharge 2 weeks later, 18 months follow-up, unchanged
17	Lee SJ, et al	2012	55	F	Resection, Conservative	Not mentioned	47 months follow-up, asymptomatic
18	Radoi MP, et al	2012	45	F	Myxoma 1 year ago, Craniotomy twice for 2 lesions	Minor neurological deficits	Discharge 3 weeks later, 12 months follow-up, unchanged
19	Chiang KH, et al	2011	52	F	Resection, Conservative	None	2 years follow-up, unchanged
20	Eddleman CS, et al	2010	18	M	Resection, Conservative	Not mentioned	4 months follow-up, multiple aneurysms formated and hemmorrhage, 3 months later, several aneurysms enlarged and hemmorrhage
21	Koo YH, et al	2009	65	F	Resection, Conservative	None	6 months follow-up, unchanged
22	Shinn SH, et al	2009	48	F	Resection, Conservative	Complex-focal type of status epilepticus	Dead due to sepsis 22 days after surgery
23	Ryou KS, et al	2008	27	F	Resection, Conservative	Intermittent headache	11 years follow-up, unchanged
24	Li Q, et al	2008	27	F	Resection, Conservative	None	2 years follow-up, unchanged
25	Kvitting JP, et al	2008	55	F	Resection, Conservative	None	6 months follow-up, unchanged
26	Sedat J, et al	2007	50	F	Resection, None	None	5 years later aneurysms formated and radiation therapy, 1 year follow-up, one aneurysm regressed
27	Namura O,et al	2007	35	M	Resection, Conservative	Raynaud’s phenomenon	10 years follow-up, unchanged
28	Herbst M, et al	2005	31	M	Resection, Conservative	None	2 years follow-up, unchanged
29	Sabolek M, et al	2005	43	F	Resection, Conservative	None	15 months follow-up, one aneurysm regressed
30	Chen Z, et al	2005	19	F	Resection, None	None	2 years later multiple aneurysms formated and conservative therapy, 1 year follow-up, unchanged
31	Josephson SA, et al	2005	33	F	Not mentioned	Not mentioned	8 years follow-up, unchanged
32	Ashalatha R, et al	2005	54	M	Resection, None	None	6 months follow-up, multiple aneurysms formated and Conservative therapy
33	Altundag MB, et al	2005	41	F	Resection, Radiation 1 year later	None	4 years follow-up, unchanged
34	Stock K, et al	2004	22	F	Resection 2 times, conservative	None	11 years follow-up, some aneurysms regressed and some aneurysms smaller, no new aneurysm
35	Yilmaz MB, et al	2003	38	F	Recurrence and resection of myxoma, coil embolization for one giant aneurysm	None	Follow-up, unchanged
36	Furuya K, et al	1995	35	M	Resection, Conservative	None	19 months follow-up, enlarged and craniotomy, another 5 months follow-up, unchanged
37	Mattle HP, et al	1995	49	M	Resection, Conservative	Not mentioned	5 years follow up, aneurysm formated 3 years after surgery, demented 5 years later and continuously progressed
38	Suzuki T, et al	1994	34	M	Resection, Conservative	Not mentioned	Follow-up, aneurysm formated 2 months after surgery, and enlarged 5 months later
39	Chen HJ, et al	1993	68	F	Resection, Conservative	Not mentioned	Craniotomy 1 year later, 2 years follow-up, unchanged
40	Hung PC, et al	1992	10	F	Resection, Conservative	None	8 months follow-up, unchanged
41	Bobo H, et al	1987	15	F	Resection four times for recurrent myxoma, Conservative	None	6 months follow-up, unchanged
42	Reed OM, et al	1986	25	F	Resection, Conservative	Not mentioned	12 years follow-up, clip for a large aneurysm 9 years later
43	Branch CL, et al	1985	53	F	Resection, Conservative	None	18 months follow-up, one aneurysm disappeared
44	Leonhardt ET, et al	1977	31	M	Resection, Conservative	None	2 months follow-up, unchanged
45	Damásio H, et al	1975	43	F	Resection, Conservative	None	1 year follow-up, unchanged
46	Steinmetz EF, et al	1973	48	F	Conservative, Conservative	SAH and hematoma evacuation	Dead 2 months later, autopsy verified myxoma with cerebral aneurysms
47	Burton C, et al	1970	41	F	None, craniotomy first	Not mentioned	Dead in the first day after surgery
48	New PF, et al	1970	41	F	Resection, Conservative	None	8 years follow-up, unchanged
49	Price DL, et al	1970	21	F	Conservative, Conservative	Not mentioned	Dead 11 months later, autopsy verified myxoma with cerebral aneurysms
50	Stoane L, et al	1966	29	M	Resection, Conservative	None	2 months follow-up, slightly larger and conservative therapy

There are no clinical practice guidelines on such patients. Myxoma was suggested to be resected first to prevent systemic emboli and mitral valve obstruction [1, 10]. In the meantime, fusiform aneurysm is not suitable for clipping or coiling compared to saccular aneurysm, surgical procedure is still an important intervention [60]. Fortunately, the SAH rate of multiple cerebral fusiform aneurysms related to atrial myxoma was low [27]. In addition, it is reported that the cerebral aneurysms regressed after myxoma resection in some cases [3, 5]. Therefore, a conservative treatment approach for cerebral aneurysms was recommended by the preoperative MDT meeting.

Anesthesia management was an enormous challenge. Few piece of evidence was found in the database to guide optimal clinical anesthesia practice. The procedural strategy was to prevent ischemic and hemorrhagic stroke. Intraoperative cerebrovascular monitoring techniques remain controversial [61]. PbtO_2_ monitoring was recommended to detect brain ischemia and intracranial hypertension in neurocritical care patients [62]. As is known to all, the transmural pressure (TMP) of cerebral aneurysm is equal to cerebral perfusion pressure (CPP), which depends on mean arterial pressure (MAP) and intracranial pressure (ICP).

$$ \mathrm{TMP}=\mathrm{CPP}=\mathrm{MAP}-\mathrm{ICP} $$

Therefore, an increase in MAP or a decrease in ICP will lead to an increase in CPP, which might increase the risk of rupture of aneurysm. On the contrary, a decrease in MAP or an increase in ICP will increase the risk of cerebral ischemia [63]. Firstly, induction of general anesthesia was an important step. One patient was reported to develop an acute left hemiparesis during induction [5]. Thus, it is crucial to control the TMP diligently. MAP and heart rate (HR) was recommended to close to baseline [64]. Lidocaine is beneficial to such patients, which could not only blunt cerebral hemodynamic response to endotracheal intubation, but also attenuate proinflammatory effects [65, 66]. Besides, esmolol and fentanyl were demonstrated to prevent hemodynamic fluctuation related to intubation in a randomized controlled trial [67]. Secondly, cardiopulmonary bypass (CPB) is a risk factor of stroke, whose pathophysiological mechanisms refer to hemorrhagic, global ischemia, and embolic [68]. TEE plays a vital role in evaluating embolism originated from the heart [69]. On the other hand, it is instrumental to detect the pathogenesis of hypotension, guide fluid replenishment and identify mitral regurgitation and shunt flow [70]. With respect to SAH, perioperative hypertension and anticoagulation are common in the cardiac surgery [68], which may increase the risk of aneurysm rupture. Although a most recent large observational study investigated the risk of postoperative 30-day SAH was 0.29%, not higher than general population [71], it was suggested to decrease CPB time and intensively control the blood pressure [68]. In addition, PaCO_2_ should be maintained at normal level, and hyperventilation, which will decrease ICP, should be avoided [72]. In this case, the CPB time was 41 min, fluctuation range of MAP was within 10%, and PaCO_2_ was normal throughout the procedure. Thirdly, the fast track cardiac anesthesia was implemented to evaluate neurological function early after procedure, which aims to extubation within 1 ~ 6 h post-operation [73]. However, tracheal extubation should be paid more attention, when tachycardia, hypertension and coughing frequently occur [74]. And it would increase the risk of aneurysm rupture. Fentanyl attenuates cough and cardiovascular response effectively, which can be safely used in fast track cardiac anesthesia [75–77]. Fortunately, refined perioperative anesthesia management was performed in this rare case, and the patient recovered uneventfully.

Atrial myxoma-related cerebral aneurysms are always multiple and in a fusiform shape in most occasions. Early resection of myxoma and conservative therapy of aneurysm is an optimal treatment. It is a great challenge to anesthesiologists to prevent stroke perioperatively. TEE and PbtO_2_ monitoring play an essential role in anesthesia management. Fast track cardiac anesthesia is safe and effective to early evaluate neurological function. Long term follow-up for “myxomatous aneurysms” is recommended. And outcome of most patients is excellent. Further study is needed to reveal the mechanism of atrial myxoma resulting in multiple cerebral aneurysms.

## Data Availability

The datasets used and analysed during the current study are available from the corresponding author on reasonable request.

## Abbreviations

AMR: Atrial myxoma resection
MCAs: Multiple cerebral aneurysms
CTA: Computed tomography angiography
TTE: Transthoracic echocardiography
MDT: Multidisciplinary team
HR: Heart rate
MAP: Mean arterial pressure
TEE: Transesophageal echocardiography
PbtO2: Parenchymal brain oxygen
ICU: Intensive care unit
NRS: Numeric rating scale
SAH: Subarachnoid hemorrhage
IL-6: Interleukin-6
TMP: Transmural pressure
CPP: Cerebral perfusion pressure
ICP: Intracranial pressure
CPB: Cardiopulmonary bypass
